# Toward trustworthy virtual cells: a roadmap for perturbation-resolved, context-aware, and experimentally validated cell models

**DOI:** 10.3389/fcell.2026.1900624

**Published:** 2026-09-08

**Authors:** Wangshu Li, Aziz Ur Rehman Aziz, Bowen Xu, Jingshan Xu, Xiaohui Yu, Daqing Wang, Chunfang Ha

**Affiliations:** 1 Key Laboratory of Biotherapy, Dalian Women and Children’s Medical Center Group, Women and Children’s Hospital Affiliated to Dalian University of Technology, Dalian, Liaoning, China; 2 Bengbu Medical University, Bengbu, China; 3 General Hospital of Ningxia Medical University, Yinchuan, China

**Keywords:** model validation, multimodal integration, single-cell perturbations, trustworthy AI, virtual cells

## Abstract

Single-cell perturbation technologies, multimodal omics, spatial profiling, and generative modeling are transforming the virtual cell from a theoretical concept into a practical objective for cell biology. Yet current efforts often emphasize model scale, data volume, or predictive breadth, while the trustworthiness required for scientific and translational use remains insufficiently addressed. We argue that virtual-cell models become decision-relevant only when the evidential chain connecting data, model design, benchmarking, and experimental validation is made explicit. We introduce the trustworthy virtual cell, a perturbation-resolved, context-aware, and experimentally validated system capable of supporting biological inference, experimental design, and preclinical decision-making. We organize recent progress around four empirical layers, namely, molecular cell state, intervention, biological context, and orthogonal phenotype, complemented by structured priors. This framework explains why static atlases, transcriptome-only readouts, and in-distribution benchmarks are insufficient for predicting cellular behavior under new conditions. Across mechanistic, deep generative, foundation, and hybrid models, we discuss trade-offs among interpretability, scalability, and extrapolation. We further argue that evaluation should move beyond held-out reconstruction accuracy toward biologically meaningful criteria, including generalization to unseen cell types and perturbations, dose, time, and combination response extrapolation, uncertainty calibration, and mechanistic consistency. We then propose a closed-loop validation ladder connecting *in silico* predictions to CRISPR perturbation, imaging, organoid, and tissue-level assays. Our aim is to review what current models can and cannot yet do, and to set out an evidential framework specifying what a virtual cell must demonstrate before its outputs are acted upon, noting which requirements are attainable today and which remain longer-term goals.

## Introduction

The cell is the smallest biological system that can be measured, perturbed and, increasingly, simulated. Yet most biological inference still proceeds by assembling local observations into narratives rather than by testing hypotheses in executable models of cell state. The virtual cell emerged as an attempt to close this gap: a computational entity that integrates molecular knowledge, cellular context and perturbational logic into a model that can be queried, falsified and updated *in silico* before or alongside wet-lab experimentation (Loew and Schaff, 2001; Karr et al., 2012; Johnson et al., 2023; Bunne et al., 2024). Early efforts established the ambition through reaction–diffusion platforms, mechanistic network models and whole-cell simulations with strong biophysical interpretability (Loew and Schaff, 2001; Karr et al., 2012; Johnson et al., 2023). They demonstrated that cellular behaviour can, in principle, be formalized. They also revealed the classical bottlenecks of the field: incomplete observability, laborious parameterization, brittle assumptions and poor portability across contexts (Karr et al., 2012; Johnson et al., 2023; Bunne et al., 2024). The past several years have changed the practical meaning of the virtual cell. Single-cell multi-omics, spatial transcriptomics, proteogenomics, pooled genetic and chemical perturbation screens, and image-based phenomics now provide cellular measurements across modality, time and context (Ma et al., 2026; Rozenblatt-Rosen et al., 2017; Rood et al., 2024a; Peidli et al., 2024; Ashuach et al., 2023; Gayoso et al., 2021; Lopez et al., 2018; Liu et al., 2025; Klein et al., 2025; Chandrasekaran et al., 2024; Dixit et al., 2016; Srivatsan et al., 2020; Feldman et al., 2022; Kudo et al., 2025). In parallel, deep generative modeling, neural optimal transport and the first generation of biological foundation models have made it possible to learn structured cellular representations directly from data rather than hand-specifying every mechanism *a priori* (Ashuach et al., 2023; Gayoso et al., 2021; Lopez et al., 2018; Liu et al., 2025; Klein et al., 2025; Chandrasekaran et al., 2024; Jumper et al., 2021; Lotfollahi et al., 2019; Bunne et al., 2023; Lotfollahi et al., 2023; Roohani et al., 2024; Piran et al., 2024; Cui et al., 2024; Hao et al., 2024; Yang et al., 2024; Wei et al., 2026). Recent studies show that such models can integrate partially paired modalities, align cells through time and space, predict transcriptional responses to genetic and chemical perturbations, simulate disease-associated state transitions and nominate candidate therapeutics (Ashuach et al., 2023; Gayoso et al., 2021; Lopez et al., 2018; Liu et al., 2025; Klein et al., 2025; Chandrasekaran et al., 2024; Jumper et al., 2021; Lotfollahi et al., 2019; Bunne et al., 2023; Roohani et al., 2024; Piran et al., 2024; Cui et al., 2024; Hao et al., 2024; Yang et al., 2024; Wei et al., 2026; Zheng et al., 2025).

**FIGURE 1:**
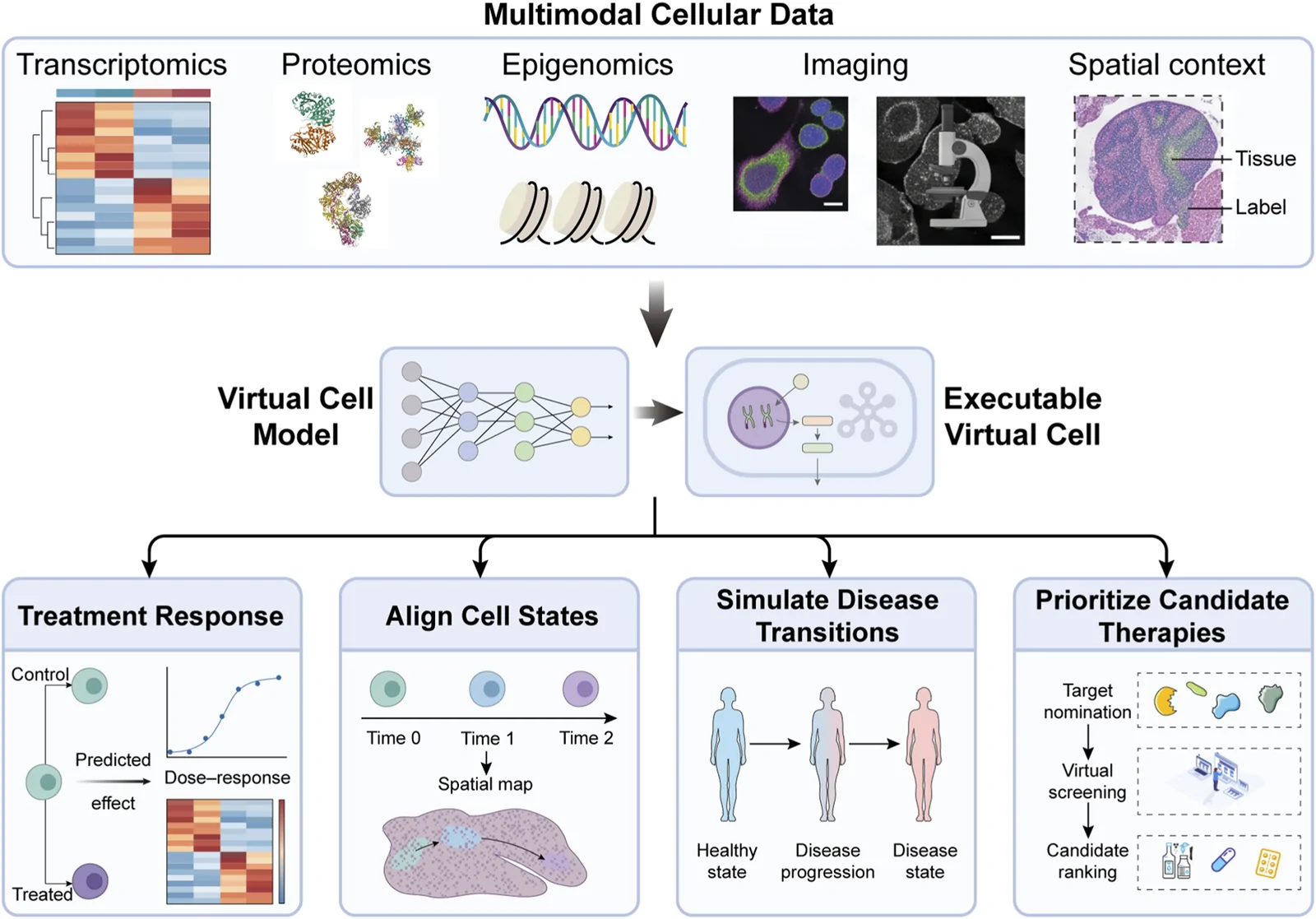
Multimodal molecular, imaging, and spatial data are integrated into virtual-cell models to support treatment-response prediction, temporal and spatial state alignment, disease-transition modeling, and therapeutic candidate prioritization..

This progress, however, should not be mistaken for a solved problem. Greater predictive capacity has not yet produced a trustworthy virtual cell (Figure 1). Much of the current literature still evaluates models within individual datasets and under task formulations that do not adequately test generalization across laboratories, platforms, cell states or perturbation regimes (Qian et al., 2025; Roohani et al., 2025; Wu, 2026; Liu et al., 2025; Wei et al., 2026; Ahlmann-Eltze et al., 2025; Luecken et al., 2025). Recent large-scale benchmarking makes this limitation explicit: when assessed in unseen contexts or against new perturbations, advanced models often show smaller gains than expected and may not consistently outperform strong baselines (Wei et al., 2026; Ahlmann-Eltze et al., 2025). Performance metrics are also frequently misaligned with the questions biologists and translational researchers actually ask. Dose, time, combinatorial perturbations and multicellular context remain underrepresented; uncertainty is inconsistently quantified; mechanistic faithfulness is rarely measured directly; and orthogonal validation is still not standard practice (Ma et al., 2026; Qian et al., 2025; Roohani et al., 2025; Wu, 2026; Chandrasekaran et al., 2024; Dixit et al., 2016; Srivatsan et al., 2020; Feldman et al., 2022; Kudo et al., 2025; Wei et al., 2026; Zheng et al., 2025; Replogle et al., 2022; Gangwal and Lavecchia, 2025) (Figure 1). A model that reconstructs held-out expression profiles well may still fail when asked to guide an experiment, discriminate mechanisms or prioritize a therapeutic hypothesis.

We therefore argue that the next bottleneck is not simply model scale, but model credibility. Taken together, the preceding evidence supports a central hypothesis for this Review: current virtual-cell models fail to become decision-relevant not primarily because they are too small, but because the evidential chain linking data, model, benchmark and validation remains fragmented. In practice, failures in generalization and mechanistic utility arise when perturbation-resolved data, contextual support, uncertainty estimation and falsification are treated as optional add-ons rather than as coupled requirements (Bunne et al., 2024; Ma et al., 2026; Qian et al., 2025; Roohani et al., 2025; Wu, 2026; Wei et al., 2026; Ahlmann-Eltze et al., 2025; Luecken et al., 2025; Gangwal and Lavecchia, 2025). We therefore hypothesize that a trustworthy virtual cell emerges only when multimodal, context-aware and perturbation-resolved data are coupled to support-aware model design, out-of-distribution benchmarking and closed-loop experimental challenge (Figure 1). In this Review, we use the term trustworthy virtual cell to denote a model that satisfies five criteria: it is built on a context-aware data foundation; it generalizes beyond the training distribution; it provides interpretable or mechanistically faithful explanations commensurate with its deployment claim; it reports calibrated uncertainty; and it can be challenged and improved through closed-loop experimentation (Bunne et al., 2024; Ma et al., 2026; Qian et al., 2025; Roohani et al., 2025; Wu, 2026; Wei et al., 2026; Ahlmann-Eltze et al., 2025; Luecken et al., 2025; Gangwal and Lavecchia, 2025). This definition shifts the field’s center of gravity from model construction to model validation. It also reframes the value proposition of virtual cells. The goal is not to replace experiments with opaque prediction, but to compress complexity into experimentally testable hypotheses.

## Data foundations for trustworthy virtual cells

**FIGURE 2:**
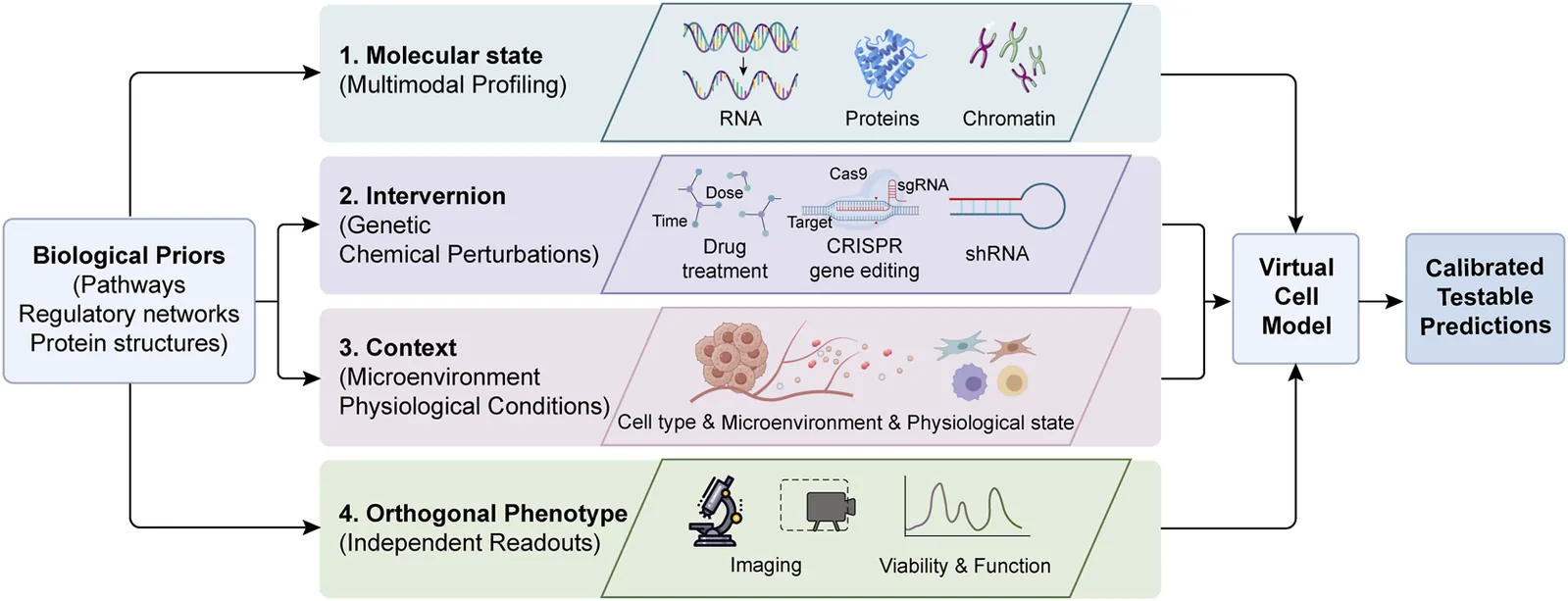
Data foundations for trustworthy virtual cells. Four empirical layers molecular state, intervention, biological context, and orthogonal phenotype together with structured biological priors provide the data foundation for context-aware and experimentally testable virtual-cell predictions.

A trustworthy virtual cell is limited by the empirical support of the data on which its counterfactuals are defined. This requirement is stricter than the familiar claim that more data are always better. For a model to support decisions rather than merely interpolate within a familiar dataset, its data foundation must jointly specify four empirical layers, molecular state, intervention, context and orthogonal phenotype, supplemented by a fifth layer of structured biological priors (Figure 2) (Bunne et al., 2024; Ma et al., 2026; Qian et al., 2025; Roohani et al., 2025; Wu, 2026; Rozenblatt-Rosen et al., 2017; Rood et al., 2024a; Peidli et al., 2024). The central question is therefore not scale alone, but whether the data make the relevant counterfactuals identifiable and falsifiable.

### Perturbation-resolved molecular state spaces

The first empirical layer is a representation of molecular state at sufficient resolution to distinguish relevant cell identities and trajectories. Static reference atlases, including the Human Cell Atlas, provide the descriptive backbone of the field (Rozenblatt-Rosen et al., 2017; Rood et al., 2024b). They define where cells sit in expression space and how many states a model must represent. Yet a static census is not enough to build a virtual cell in any strong sense. A model cannot learn the semantics of intervention from observational atlases alone. It must observe how states move under perturbation, and how those movements depend on dose, duration and starting context (Rood et al., 2024a; Peidli et al., 2024; Dixit et al., 2016; Srivatsan et al., 2020).

Perturbation-resolved single-cell profiling has therefore become the decisive substrate for modern virtual-cell efforts. Perturb-seq linked pooled CRISPR perturbation with single-cell transcriptomics and turned circuit dissection into a scalable state-mapping problem (Dixit et al., 2016; Datlinger et al., 2017; J et al., 2016; Adamson et al., 2016; Schraivogel et al., 2020; Replogle et al., 2020). Sci-Plex extended this logic to chemical perturbations and dose-response profiling, opening the door to large-scale pharmacologic response modeling (Srivatsan et al., 2020). Curated resources such as scPerturb now aggregate transcriptomic, proteomic and epigenomic perturbation datasets in a harmonized format, reducing a major bottleneck in method development and benchmarking (Peidli et al., 2024). The field is thus moving from isolated perturbation studies toward perturbation-ready data ecosystems.

For trustworthy virtual-cell construction, the critical issue is not only whether perturbation data exist, but whether the intervention axis is richly annotated. Perturbation identity, modality, dose, time, replication structure, batch and cellular context all matter. Missing these variables does not merely reduce statistical power; it changes the biological meaning of the prediction target. Under-annotated screens can make batch effects look biological.

### Multimodal measurements and the problem of partial pairing

Transcriptomes alone do not exhaust cell state. Protein abundance, chromatin accessibility, lineage relationships and post-transcriptional regulation all shape cellular behaviour, and many perturbations act first through layers not visible in RNA readouts (Ashuach et al., 2023; Gayoso et al., 2021; Lopez et al., 2018; Liu et al., 2025; Jumper et al., 2021; Hao et al., 2021; Argelaguet et al., 2020). Multimodal single-cell technologies and the models built around them are therefore central to the virtual-cell agenda. TotalVI and related frameworks showed that joint probabilistic modeling can connect RNA and protein measurements in a shared latent space (Gayoso et al., 2021). MultiVI generalized this logic to chromatin and transcriptomic modalities, including settings with only partial pairing across assays (Ashuach et al., 2023). Benchmarking work now makes clear that multimodal integration is itself a nontrivial methodological problem with strong consequences for downstream biological conclusions (Liu et al., 2025; Luecken et al., 2022; Wang H et al., 2025; Rautenstrauch and Ohler, 2025; Maan et al., 2024; Zhang et al., 2025).

Partial pairing deserves special emphasis. In realistic experimental programs, some cells are measured across multiple modalities, whereas others are available only in one assay. A model that treats all modalities as fully paired may overstate certainty and import assay-specific artifacts into the latent space. Conversely, a model that ignores partial pairing wastes information. Trustworthy virtual cells therefore require explicit treatment of missingness, uneven support and measurement asymmetry. The goal is not to force a perfectly unified latent representation, but to learn one whose confidence reflects how much cross-modal evidence is actually present.

Multimodality also changes what counts as validation: paired protein and chromatin measurements can test whether predicted transcriptomic trajectories are reflected in orthogonal molecular layers.

### Spatial, temporal, physiological and population context

Cells do not act in isolation. State transitions depend on developmental history, physical location, neighboring cell types and extracellular signals (Klein et al., 2025; Biancalani et al., 2021; Lopez et al., 2019; Li et al., 2022; La Manno et al., 2018; Bergen et al., 2020; Schiebinger et al., 2019; Setty et al., 2019; Wolf et al., 2019; Longo et al., 2021; Dries et al., 2021; Palla et al., 2022; Browaeys et al., 2020; Jin et al., 2021). A trustworthy virtual cell therefore requires more than a molecular snapshot; it requires information about where a cell is, where it came from and what it is exposed to. Spatial transcriptomics, longitudinal sampling and lineage-aware measurements each contribute to this goal, but they solve different parts of the problem. Context also includes donor- and population-level variables such as age, sex, chronic disease, genetic background or ancestry, geographic and environmental exposure, and physiological status. These variables should be recorded as metadata and, when relevant to the deployment claim, used as explicit conditioning or holdout axes. Ethnicity and geography should be treated as imperfect proxies for ancestry and exposure rather than as intrinsic cellular labels (Rozenblatt-Rosen et al., 2017; Rood et al., 2024b; The Tabula Sapiens Consortium et al., 2022).

Time-series methods such as RNA velocity and dynamical extensions estimate local movement in state space, while optimal-transport approaches provide a more global view of transitions between populations or time points (La Manno et al., 2018; Bergen et al., 2020; Schiebinger et al., 2019; Setty et al., 2019; Wolf et al., 2019). Moscot extends this logic by mapping cells jointly through time and space, providing a useful model for how transport-based methods can link developmental or disease progression to tissue organization (Klein et al., 2025).

Spatial context adds a second dimension of constraint. Tools such as Tangram, Giotto and Squidpy enable alignment between single-cell and spatial data or the interrogation of local tissue structure (Biancalani et al., 2021; Li et al., 2022; Dries et al., 2021; Palla et al., 2022). Ligand–receptor models such as NicheNet and CellChat further connect spatial neighbourhoods to putative communication programs (Browaeys et al., 2020; Jin et al., 2021). Spatially aware foundation models such as Nicheformer suggest that context-sensitive representation learning will increasingly operate across both molecular and tissue scales (Tejada-Lapuerta et al., 2025). The consequence for virtual cells is clear: predictions that ignore microenvironmental context may be acceptable for some cell-intrinsic tasks, but they are insufficient for questions involving immune signaling, stromal interactions, morphogenesis or tissue remodeling.

### Orthogonal phenotypes: imaging, morphology and function

A transcriptome is an informative readout, but it is also a narrow one. If both training and evaluation remain confined to transcriptomic similarity, virtual-cell models risk self-confirmation: they can look convincing because they reproduce the only modality they were trained to predict. Orthogonal phenotypes help break this loop. Imaging, morphology, proteomic function, electrophysiology or simple viability measurements each provide external views of the same underlying state, and together they test whether a predicted perturbational effect reflects biology rather than transcriptomic pattern matching (Chandrasekaran et al., 2024; Feldman et al., 2022; Kudo et al., 2025; Bray et al., 2016; Way et al., 2022).

Image-based profiling is especially important because it can be scaled, pooled and matched to perturbation data. Cell Painting established a high-content assay for multiplex morphological profiling (Bray et al., 2016), and large matched resources such as CPJUMP1 now connect chemical and genetic perturbations to millions of images and morphological profiles (Chandrasekaran et al., 2024). Recent methods extend image-based pooled screening to primary cells and tissue settings (Feldman et al., 2022; Kudo et al., 2025). These datasets matter because they provide phenotypes not trivially reducible to RNA abundance and closer to the way many screens are judged in practice.

Orthogonal phenotypes also offer a more stringent basis for mechanism-of-action analysis. If a predicted perturbational rescue is accompanied by concordant movement in morphology, pathway activity and viability, the claim is stronger than if it is supported by expression similarity alone. Trustworthy virtual cells should therefore be trained and evaluated, whenever possible, against at least one phenotype that does not share the same measurement channel as the primary target.

### Structured priors and biological knowledge as a fifth layer

A fifth layer of support comes from structured biological priors: protein structures, pathway databases, regulatory networks, interaction graphs and drug–target knowledge (Jumper et al., 2021; Lamb et al., 2006; Subramanian et al., 2017; Wishart et al., 2018; The Gene Ontolo gy Consortium, 2021; Gillespie et al., 2022; Szklarczyk et al., 2023). These priors matter because cellular data are sparse, noisy and biased toward the measured conditions. Knowledge can regularize learning, improve sample efficiency and constrain explanation. Reactome, Gene Ontology, STRING and DrugBank provide reusable biological scaffolds for pathway- or target-level interpretation, whereas structural resources such as AlphaFold expand the prior layer available to model design (Jumper et al., 2021; Wishart et al., 2018; The Gene Ontolo gy Consortium, 2021; Gillespie et al., 2022; Szklarczyk et al., 2023).

Yet priors must be treated as support, not as substitute evidence. A pathway graph cannot make an unsupported extrapolation suddenly reliable. Nor should biologically plausible explanations be accepted simply because they align with known pathways. Knowledge-informed models such as GeneCompass, scKGBERT and other network-aware architectures are promising precisely because they try to integrate priors with data rather than replace data with prior belief (Yang et al., 2024; Li et al., 2025; Bahrami et al., 2026). The trustworthy use of prior knowledge therefore depends on the same principle that governs every other part of the virtual-cell stack: it must sharpen falsifiable predictions rather than merely decorate them.

Taken together, these considerations suggest a simple rule. A decision-relevant virtual cell requires a data stack that jointly specifies what state is measured, what intervention is applied, in what context it occurs, and by which orthogonal phenotypes it can be tested. Structured priors can guide learning and interpretation, but they cannot rescue a model that lacks empirical support for the counterfactuals it claims to predict.

## Model classes and their trade-offs

No single model class currently solves the virtual-cell problem. Instead, the field is organized around a set of families that make different trade-offs between interpretability, scalability and extrapolation (Bunne et al., 2024; Ma et al., 2026; Qian et al., 2025; Roohani et al., 2025; Wu, 2026; Cui et al., 2024; Hao et al., 2024; Yang et al., 2024; Wei et al., 2026; Fu et al., 2025). Those trade-offs should be explicit, because the best model for one deployment claim can be the wrong model for another. A model designed for mechanistic hypothesis generation is not judged by the same criteria as a model designed for broad representation learning across millions of cells.

### Mechanistic and rule-based models: explicit causality, limited scale

Mechanistic and rule-based models remain the most interpretable branch of the field. The Virtual Cell platform, whole-cell simulations and related reaction–diffusion or network-based approaches define variables and interactions with explicit biological meaning (Loew and Schaff, 2001; Karr et al., 2012; Johnson et al., 2023). This makes them attractive for causal reasoning, parameter sensitivity analysis and hypothesis testing under clearly stated assumptions. For well-characterized processes, mechanistic models still provide the clearest path from prediction to explanation.

Their limitations are also well known. Mechanistic coverage is expensive to build, parameterization is difficult, and transferring a model across cell types or conditions often requires extensive re-specification (Karr et al., 2012; Johnson et al., 2023; Bunne et al., 2024). These models also struggle with the scale and heterogeneity of modern single-cell data. For this reason, purely mechanistic approaches are unlikely to become the dominant architecture for atlas-scale virtual cells. Their continuing importance lies elsewhere: as interpretable components, as sources of inductive bias and as benchmarks for whether statistical models recover biologically reasonable behaviour.

### Deep generative perturbation models: flexible counterfactuals, implicit mechanisms

The first major modern wave of virtual-cell modeling came from deep generative models trained on single-cell data. scVI established a variational framework for learning latent cellular structure from noisy count data (Lopez et al., 2018). scGen then showed how such representations could be used to predict perturbation responses as vector-like movements through latent space (Lotfollahi et al., 2019). Subsequent methods expanded the space of counterfactual modeling. CellOT formulated perturbational mapping as optimal transport (Bunne et al., 2023); CPA modeled compositional responses to combinations of perturbations and covariates (Lotfollahi et al., 2023); GEARS explicitly targeted multigene perturbations (Roohani et al., 2024); and biolord emphasized disentanglement of biological and nuisance covariates (Piran et al., 2024).

These models are strong because they are flexible, data-driven and naturally compatible with single-cell perturbation datasets. They can interpolate within complex response manifolds and often provide useful counterfactual forecasts for unseen combinations or partially supported contexts. Their central weakness is that mechanism is usually implicit rather than enforced. A model can learn a useful latent transformation without representing the underlying regulatory or signaling logic in a form that biologists can readily interrogate. This does not make such models unscientific. It does, however, limit the strength of the mechanistic claims that can be drawn from them. For decision support, they also require careful benchmarking under support-aware splits. Without that, flexible latent models may appear more general than they really are (Wei et al., 2026; Ahlmann-Eltze et al., 2025).

### Foundation models: transfer at scale, but not yet virtual cells by default

Biological foundation models aim to learn reusable cellular representations from very large corpora of single-cell data (Cui et al., 2024; Hao et al., 2024; Tejada-Lapuerta et al., 2025; Fu et al., 2025; Boiarsky et al., 2024; Zeng et al., 2025; Kalfon et al., 2025; Kedzierska et al., 2025). scGPT, scFoundation, CellFM, scPRINT, search-oriented atlas models and related architectures show that large-scale pretraining can improve retrieval, annotation and transfer across datasets or tissues (Cui et al., 2024; Hao et al., 2024; Zeng et al., 2025; Kalfon et al., 2025; Rood et al., 2024b). Knowledge-informed variants such as GeneCompass and scKGBERT suggest that priors can help organize these representations in biologically meaningful ways (Yang et al., 2024; Li et al., 2025).

The attraction of foundation models is straightforward: scale, transfer and the possibility of unifying cellular measurements across studies, modalities and even species (Yang et al., 2024; Tejada-Lapuerta et al., 2025; Fu et al., 2025; Zeng et al., 2025; Rood et al., 2024b). Yet a large model is not a virtual cell by default. Pretraining on broad cellular corpora can produce strong embeddings while leaving perturbational semantics, causal directionality and mechanistic interpretability underdeveloped (Boiarsky et al., 2024; Kedzierska et al., 2025; Wu et al., 2025). Several studies now caution that foundation-model success is highly task-dependent and that zero-shot or out-of-distribution claims require particularly careful evaluation (Boiarsky et al., 2024; Kedzierska et al., 2025). The crucial distinction is between a model that summarizes cell states well and a model that can support experimentally grounded counterfactual reasoning.

### Hybrid and knowledge-grounded architectures: the most promising middle ground

Hybrid architectures attempt to combine the coverage of data-driven models with the biological legibility of structured priors. This broad category includes knowledge-informed foundation models (Yang et al., 2024; Li et al., 2025), graph- or program-aware predictors (Theodoris et al., 2023; Wang T. et al., 2025; Chevalley et al., 2025; He et al., 2025; Wang F-a et al., 2025), and newer proposals that move from modality-specific to compositional or world-model-style systems (Wei et al., 2025; Bahrami et al., 2026). The rationale is compelling. Purely mechanistic models do not scale well, while purely data-driven models often generalize or explain less reliably than their benchmark scores suggest. Hybrid models try to capture the best of both worlds by learning from data under biologically meaningful constraints.

**FIGURE 3:**
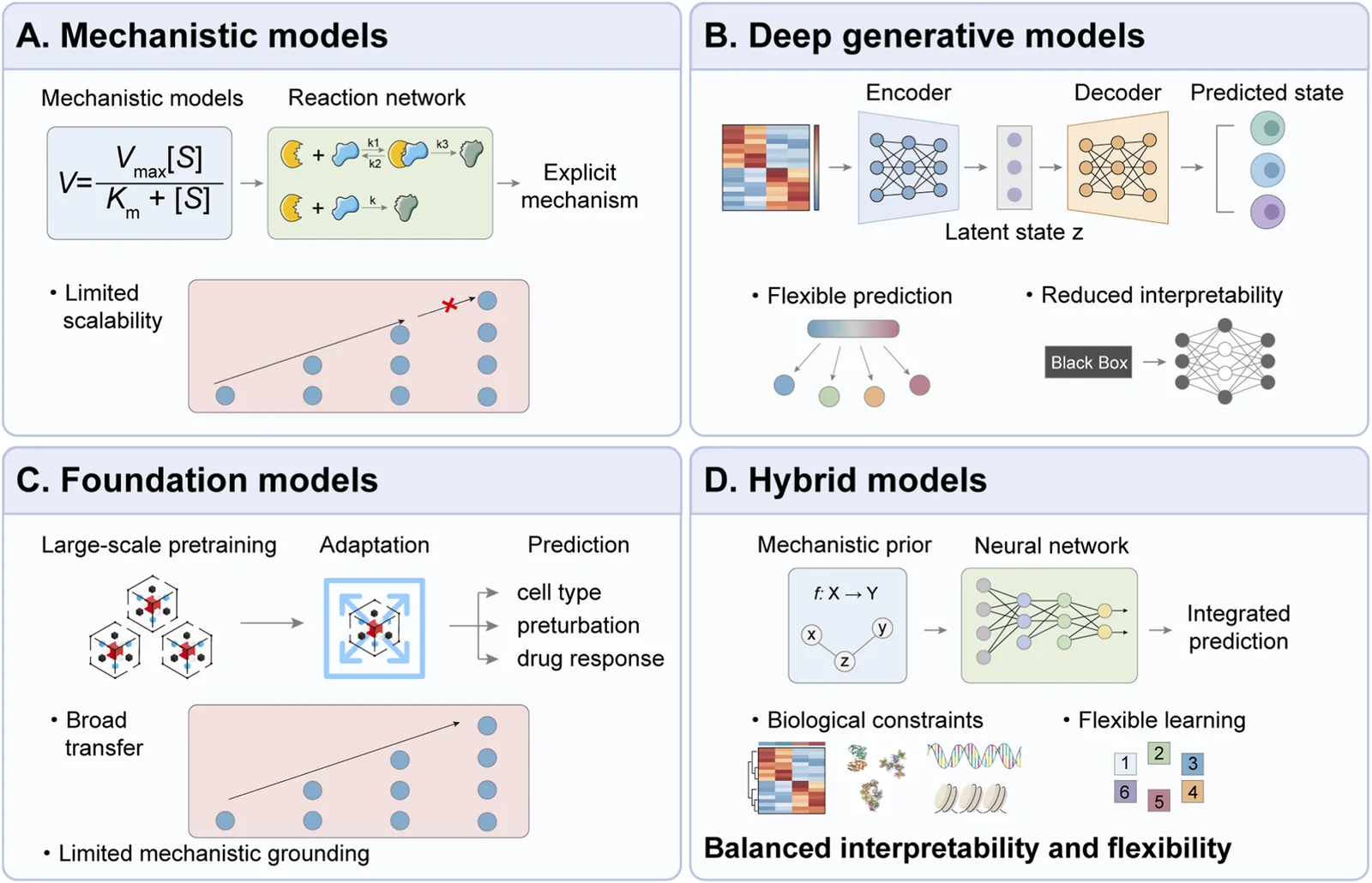
Model classes and their trade-offs in virtual-cell modeling. **(A)** Mechanistic models provide explicit biological interpretation but limited scalability. **(B)** Deep generative models enable flexible state prediction with reduced interpretability. **(C)** Foundation models support large-scale transfer across tasks and datasets. **(D)** Hybrid models combine biological constraints with data-driven learning to balance interpretability and predictive flexibility.

Bunne et al. separate a universal representation, a shared and transferable encoding of cell state, from virtual instruments, the task-specific modules that interrogate, decode or perturb that representation (Bunne et al., 2024). This decomposition is complementary to the taxonomy in Figure 3 rather than identical with it. A universal representation will often be learned by a foundation model, whereas a virtual instrument may be mechanistic, generative or hybrid. In our usage, hybrid specifically denotes architectures that couple learned representations to structured biological priors, explicit mechanisms or modular constraints, not merely the decoupling of representation from instrumentation.

Recent work illustrates several routes into this design space. CellNavi links cell-state manifolds to gene graphs and uses that structure to nominate drivers of cellular transitions (Wang T. et al., 2025). scTFBridge brings TF-motif information into multi-omic generative modeling, helping connect state prediction to regulatory control (Wang F-a et al., 2025). Network-transfer approaches show that transferable biological structure can be learned across tasks (Theodoris et al., 2023), whereas large-scale network-inference benchmarks clarify what is and is not recoverable from perturbation data alone (Chevalley et al., 2025). More speculative efforts such as VCWorld and compositional foundation models push toward broader simulation frameworks in which cells are treated as partially factorized dynamical systems (Wei et al., 2025; Bahrami et al., 2026).

Hybrid approaches are not automatically superior. Priors can be wrong, overly rigid or mismatched to the deployment setting. But conceptually they are attractive because they align with the central requirement of trustworthy virtual cells: decision-relevant prediction should be coupled to explanation and to support-aware extrapolation.

### Choosing model classes by intended use

The most useful way to compare model classes is not by asking, which is best overall, but by asking which failure mode is most acceptable for the intended use. If the goal is mechanistic dissection of a relatively well-characterized process, interpretable mechanistic or hybrid models may be preferable even at smaller scale. If the goal is broad representation learning across many tissues, foundation models are attractive, provided their role is not overstated. If the goal is perturbation forecasting in a defined experimental system, deep generative models remain the most mature option. If the goal is decision support under limited support, then calibration, uncertainty and validation strategy may matter more than architectural novelty. For bounded tasks such as annotation, reconstruction within a familiar state space, or perturbation interpolation in a well-characterized system, linear, factor, nearest-neighbour or compact mechanistic models may be sufficient and preferable. The trustworthy-virtual-cell framework is therefore not a mandate to use the largest or most multimodal architecture. Requirements should scale with the claim, the available data and the cost of error. Figure 3 maps the major model families and the deployment trade-offs they make. Table 1 distills representative case studies, from mechanistic simulation to multimodal, perturbational, spatial and disease-oriented systems, to show that current progress is modular.

**TABLE 1:** Representative empirical case studies spanning the current virtual-cell design space.

Representative study/model	System and data	Primary task	Main advance for the field	Trustworthiness contribution	Current limitation
Virtual cell (Loew and Schaff, 2001)	Reaction-diffusion and pathway-based cell simulations	Executable mechanistic simulation	Established an early software environment for formal cell-biological modeling	High auditability and explicit biological semantics	Limited coverage, hard parameterization and weak portability across contexts
Whole-cell model (Karr et al., 2012)	Genome-scale mechanistic model of *Mycoplasma* genitalium	Predict phenotype from genotype	Demonstrated that multiple cellular processes can be coupled in one executable system	Strong causal transparency within a defined organism	Organism-specific, parameter-heavy and difficult to transfer
TotalVI/MultiVI (Gayoso et al., 2021; Ashuach et al., 2023)	Partially paired RNA, protein and chromatin single-cell data	Multimodal integration and completion	Made cross-modal latent-state learning practical in realistic partially paired settings	Improves support-aware state definition across modalities	Does not by itself resolve intervention semantics or causal directionality
CPA/CellOT/GEARS (Lotfollahi et al., 2023; Bunne et al., 2023; Roohani et al., 2024)	Single-cell perturbation transcriptomics with covariates and combinations	Counterfactual perturbation-response prediction	Matured the perturbation-prediction layer of the virtual cell	Enables explicit testing on unseen perturbations or combinations within measured support	Mechanism remains partly implicit; out-of-distribution robustness is still limited
Moscot (Klein et al., 2025)	Time-resolved and spatially resolved single-cell atlases	Spatiotemporal mapping and transport	Linked cell-state transitions to time and tissue context in one framework	Strengthens context-aware trajectory reasoning	Long-horizon causality and experimental validation remain difficult
Nicheformer (Tejada-Lapuerta et al., 2025)	Spatial omics with cell-neighbourhood context	Context-sensitive representation learning	Extended foundation-style modeling into tissue microenvironments	Captures neighbourhood dependence and cell-cell context	Matched spatial-perturbation datasets remain sparse and validation is challenging
CellNavi/knowledge-grounded transition models (Wang et al., 2025)	Gene graph-enhanced single-cell manifolds	Driver-gene discovery for cell-state transitions	Linked representation learning to regulator nomination and transition control	Improves intervention-relevant explanatory structure	Regulatory claims still require orthogonal confirmation
UNAGI and disease-oriented generative models (Zheng et al., 2025)	Disease single-cell data with downstream therapeutic nomination	Cellular dynamics and *in silico* drug prioritization	Moved virtual cells toward translational triage	Couples prediction to downstream experimental challenge	Still far from decision-exclusive clinical deployment

### What should a virtual cell actually predict?

**FIGURE 4:**
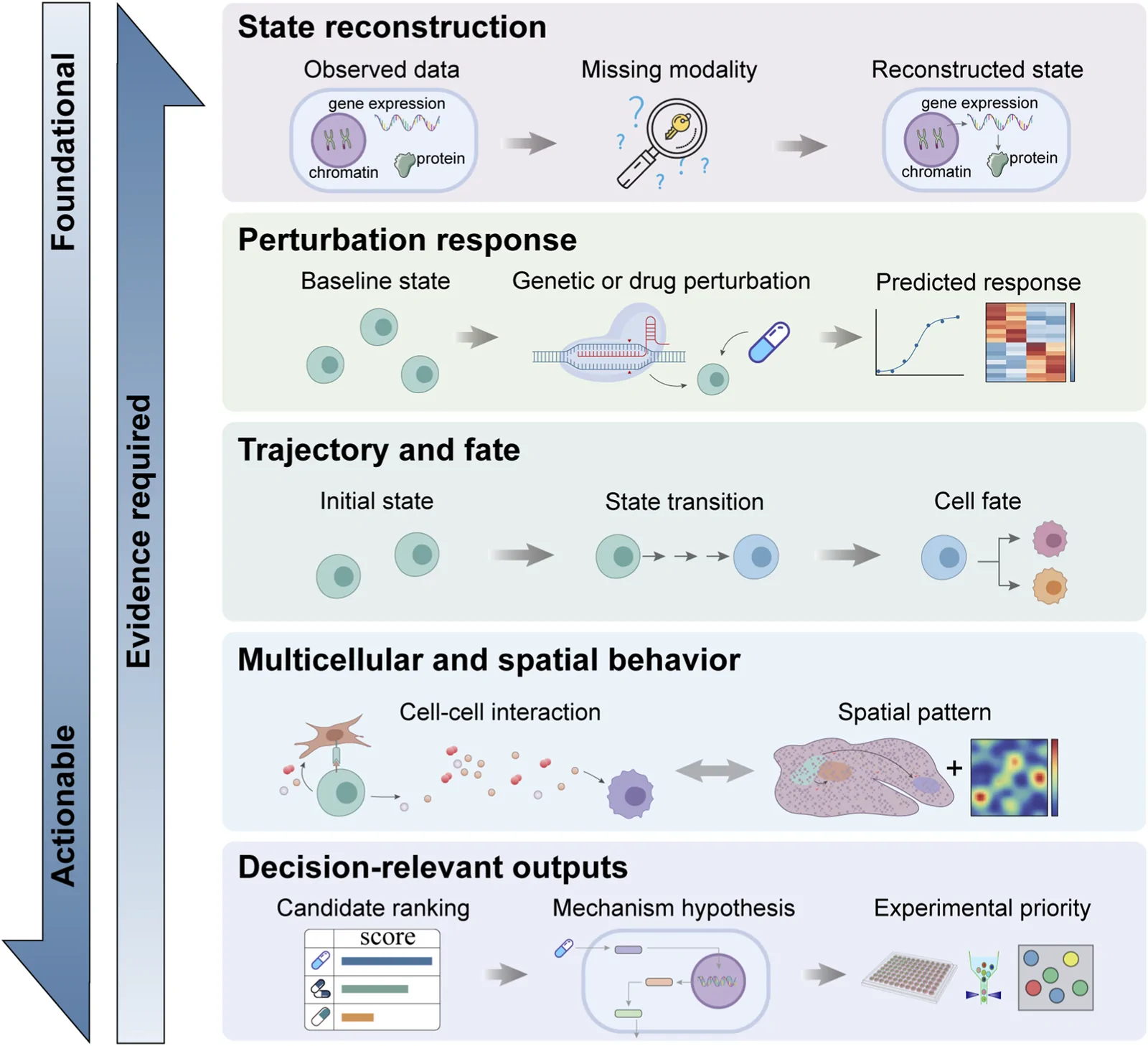
Hierarchy of virtual-cell prediction tasks. Prediction tasks progress from state reconstruction to perturbation response, trajectory and fate modeling, multicellular and spatial behavior, and decision-relevant outputs.

The phrase virtual cell often obscures a simple issue: different studies optimize different endpoints. Without clarity on the prediction target, comparisons across models become uninformative. In practice, current virtual-cell efforts can be organized around five classes of outputs: state reconstruction, perturbation response, trajectory and fate prediction, multicellular or spatial behaviour, and decision-relevant outputs such as drug ranking or mechanism-of-action inference (Bunne et al., 2024; Ma et al., 2026; Qian et al., 2025; Roohani et al., 2025; Wu, 2026; Klein et al., 2025; Chandrasekaran et al., 2024; Lotfollahi et al., 2019; Bunne et al., 2023; Lotfollahi et al., 2023; Roohani et al., 2024; Piran et al., 2024; Cui et al., 2024; Hao et al., 2024; Yang et al., 2024; Wei et al., 2026; Zheng et al., 2025; Kaul et al., 2023; Tejada-Lapuerta et al., 2025; Bao et al., 2025). These classes differ in biological ambition, evidentiary requirements and suitable metrics (Figure 4).

### State reconstruction and multimodal completion

The most conservative task is to reconstruct or impute missing measurements within a known state space. Examples include denoising transcriptomes, imputing one modality from another or completing partially paired observations (Ashuach et al., 2023; Gayoso et al., 2021; Lopez et al., 2018; Liu et al., 2025; Hao et al., 2021; Argelaguet et al., 2020). These tasks are useful because they test whether the model has learned coherent cellular representations. They also provide practical value in atlas construction and multimodal data analysis.

However, reconstruction is not equivalent to counterfactual reasoning. A model that accurately imputes held-out measurements from familiar cells may still fail under perturbation or context shift. Reconstruction tasks therefore belong at the base of the virtual-cell hierarchy, not at its summit.

### Perturbation response and counterfactual state prediction

A stronger task is to predict how a cell state changes under intervention. This includes transcriptional responses to single-gene perturbations, combinatorial perturbations, chemical treatments, dose shifts and context-dependent responses (Dixit et al., 2016; Srivatsan et al., 2020; Lotfollahi et al., 2019; Bunne et al., 2023; Lotfollahi et al., 2023; Roohani et al., 2024; Piran et al., 2024; Wei et al., 2026; Zheng et al., 2025; Replogle et al., 2022; Norman et al., 2019; Ahlmann-Eltze et al., 2025). Here the model must approximate a counterfactual: what would happen to this cell, or this cellular context, if a new intervention were applied?

This task is where the field has made the most visible progress, but it is also where evaluation can be most misleading. Predicting responses for perturbations or cell states that are already well supported by nearby training examples is biologically useful, yet very different from forecasting responses in truly novel contexts. Support structure matters. So does the granularity of the claim. Predicting average gene-expression change is easier than predicting heterogeneous responses, long-term adaptation or drug combinations.

### Trajectories, fate transitions and long-horizon dynamics

Many biological questions are intrinsically dynamical. Reprogramming, differentiation, disease progression and treatment response unfold over trajectories rather than static endpoints (Klein et al., 2025; Zheng et al., 2025; Kaul et al., 2023; Marx, 2023; La Manno et al., 2018; Bergen et al., 2020; Schiebinger et al., 2019; Setty et al., 2019; Wolf et al., 2019). A virtual cell aimed at these problems must predict not merely a new state, but a path or directional tendency. RNA velocity, optimal transport, graph abstractions and manifold-based transition models provide complementary views of this problem (Klein et al., 2025; La Manno et al., 2018; Bergen et al., 2020; Schiebinger et al., 2019; Setty et al., 2019; Wolf et al., 2019). More recent approaches such as CellNavi and disease-focused generative models move toward identifying the drivers that push cells along these trajectories (Zheng et al., 2025; Wang T. et al., 2025).

### Multicellular and spatially contextual behaviour

A further expansion is to predict cellular behaviour in microenvironmental context. This includes neighborhood-dependent signaling, tissue patterning, immune–stromal interactions and emergent multicellular organization (Klein et al., 2025; Biancalani et al., 2021; Kaul et al., 2023; Tejada-Lapuerta et al., 2025; Bao et al., 2025; Longo et al., 2021; Dries et al., 2021; Palla et al., 2022; Browaeys et al., 2020; Jin et al., 2021). Spatial alignment methods, communication models and microenvironment-aware foundation models each tackle parts of this problem (Biancalani et al., 2021; Tejada-Lapuerta et al., 2025; Browaeys et al., 2020; Jin et al., 2021). Developmental and patterning studies illustrate how even simplified “virtual microenvironment” systems can reveal behaviours that would be invisible in cell-intrinsic models (Kaul et al., 2023).

The evidentiary burden is higher here because the model must represent both cell-intrinsic and contextual determinants. Benchmarks and validation schemes must therefore distinguish between tasks that are genuinely spatial and those that merely inherit a spatial label while remaining largely cell-autonomous.

### Drug response, mechanism-of-action and decision-relevant outputs

The most ambitious task is to support action. In translational settings, this typically means ranking perturbations, nominating rescue strategies, clustering mechanism-of-action, triaging toxicity risk or prioritizing experiments (Ma et al., 2026; Chandrasekaran et al., 2024; Srivatsan et al., 2020; Kudo et al., 2025; Zheng et al., 2025; Bao et al., 2025; Gangwal and Lavecchia, 2025; Lamb et al., 2006; Subramanian et al., 2017; Wishart et al., 2018; Björnsson et al., 2020). These tasks move the virtual cell from descriptive science toward decision support. They are therefore the most attractive claims and the most dangerous to overstate.

A useful distinction is between decision-relevant and decision-exclusive outputs. A virtual cell can be decision-relevant when its forecasts help focus hypotheses, reduce experimental burden or improve prioritization. It should be considered decision-exclusive only when the model alone can justify action without additional validation. Current virtual-cell systems are not yet at that second stage. The correct near-term ambition is therefore to make them better at triage, ranking and mechanistically informed experiment design.

### A hierarchy of endpoints

These task classes form a loose hierarchy. Reconstruction and multimodal completion sit at the bottom; perturbation response and trajectory forecasting occupy the middle; context-dependent behaviour and action-oriented prediction sit at the top. The further up this hierarchy a paper’s claim extends, the stronger the requirements for support-aware benchmarking, uncertainty quantification, interpretability and external validation.

## Benchmarking beyond in-distribution accuracy

Benchmarking has become the pivotal methodological bottleneck of the field. Many current evaluations use random or weakly structured train–test splits. In single-cell settings, such splits can dramatically overestimate real-world utility because train and test data often share perturbations, batches, donors or highly similar cell states (Wei et al., 2026; Ahlmann-Eltze et al., 2025; Luecken et al., 2022; Luecken et al., 2025). Even when exact cells are held out, nearby support remains abundant. This is sufficient for interpolation benchmarks, but it is inadequate if the model is meant to generalize to new perturbations, cell types, laboratories or assay conditions.

The problem is not unique to virtual cells; it is common across machine learning. But it is particularly acute here because biological datasets are highly structured. A benchmark that holds out individual observations while retaining strong neighborhood overlap can produce leaderboard gains without testing the scientific question of interest. The result is an illusion of progress: models appear to generalize, when in practice they may only interpolate across dense local support.

### Split design is the benchmark

The most important design choice in a benchmark is therefore the split definition. For perturbation modeling, the relevant split families include unseen perturbations, unseen cell types, unseen donors or batches, unseen doses or time points, and combinations of these axes (Dixit et al., 2016; Srivatsan et al., 2020; Lotfollahi et al., 2019; Bunne et al., 2023; Lotfollahi et al., 2023; Roohani et al., 2024; Piran et al., 2024; Wei et al., 2026; Zheng et al., 2025; Replogle et al., 2022; Norman et al., 2019; Ahlmann-Eltze et al., 2025). Each split corresponds to a different biological claim. A model that predicts an unseen dose for a known drug in a familiar cell type is doing something fundamentally different from a model that forecasts an unseen perturbation in an unseen context.

This principle also applies beyond perturbation prediction. In multimodal integration, cross-platform and cross-assay transfer should be tested explicitly (Liu et al., 2025; Luecken et al., 2022; Wang H et al., 2025; Rautenstrauch and Ohler, 2025; Maan et al., 2024; Zhang et al., 2025). In spatial modeling, train–test partitions should avoid leaking nearby tissue regions or nearly identical reference states (Biancalani et al., 2021; Li et al., 2022). In foundation-model evaluation, zero-shot and few-shot claims should be separated from standard fine-tuning performance (Boiarsky et al., 2024; Kedzierska et al., 2025). The split is the operational definition of generalization.

Leakage-resistant design is especially important. Cells from the same sample, guide, treatment batch or tissue region should not be distributed across train and test sets in a way that trivializes the prediction problem. Nor should hyperparameter tuning be conducted on benchmarks that are later presented as blind evaluations. If the goal is trustworthy virtual cells, the benchmark should make overclaiming difficult rather than easy.

### Metrics must match the biological question

Metric choice can distort conclusions just as strongly as split choice. Correlation between predicted and observed expression is common and often useful, but it is not universally sufficient (Wei et al., 2026; Ahlmann-Eltze et al., 2025; Wang H et al., 2025; Rautenstrauch and Ohler, 2025). For some applications, directional recovery of differential responses matters more than global reconstruction. For others, ranking candidate interventions, preserving heterogeneity or identifying whether a model knows when it does not know may be the more relevant endpoint.

A practical benchmark should therefore report a panel of metrics aligned to the task: reconstruction fidelity for completion tasks; response ranking and differential-expression recovery for perturbation tasks; trajectory consistency for dynamical tasks; neighborhood or communication recovery for spatial tasks; and calibration metrics for decision support. The metric should also match the unit of biological decision. If an experiment is chosen based on ranked perturbations, then gene-wise Pearson correlation is, at best, an incomplete proxy.

Recent work in single-cell analysis also shows that widely used embedding metrics can mislead, especially when batch removal, imbalance or manifold distortion are involved (Wang H et al., 2025; Rautenstrauch and Ohler, 2025; Maan et al., 2024; Zhang et al., 2025).

### Baselines, ablations and support diagnostics are essential

A useful benchmark does not only test sophisticated models; it tests whether they outperform the strongest reasonable alternatives. Recent analyses show that deep models do not always exceed simple linear baselines in perturbation prediction (Ahlmann-Eltze et al., 2025). This result should not be read as a failure of the field. It is a reminder that architectural novelty is easy to overvalue when baselines are weak.

Every virtual-cell benchmark should therefore include strong simple baselines, clear ablations and diagnostics of local support. Baselines might include linear models, neighborhood averaging, nearest-neighbor transfer, factor models or pathway-aware predictors, depending on the task. Ablations should test the value of multimodal inputs, priors, pretraining, spatial context and uncertainty modules. Supporting diagnostics should state how much training evidence remains near each test example. Without these components, a model may appear to extrapolate while actually free riding on dense coverage.

Benchmarking should also separate two questions that are often conflated: how well a model fits one dataset, and how robustly the strategy transfers across datasets. The first is an engineering result; the second is closer to field-level progress.

### Calibration and uncertainty should be benchmarked, not merely mentioned

For decision support, uncertainty is not a decorative add-on. It is central to whether a virtual cell can be used responsibly. If the model cannot indicate when a prediction is weakly supported, users cannot distinguish plausible extrapolation from confident error (Ma et al., 2026; Qian et al., 2025; Roohani et al., 2025; Wu, 2026; Wei et al., 2026; Gangwal and Lavecchia, 2025; Guo et al., 2017; Lakshminarayan et al., 2017). This matters especially when models are asked to rank new perturbations or forecast outcomes in rare states, where unsupported mistakes can waste substantial experimental effort.

Benchmarking should therefore make uncertainty operational. At a minimum, evaluation should test whether predictive uncertainty tracks empirical error across perturbations, contexts and datasets. Such analyses remain underused in virtual-cell studies (Guo et al., 2017; Lakshminarayanan et al., 2017), despite being closer to how these systems would actually be used.

### Toward benchmark commons and decision-grade reporting standards

The field now has the components needed to move from *ad hoc* evaluation to benchmark commons. Harmonized resources such as scPerturb reduce data-format fragmentation (Peidli et al., 2024), software ecosystems such as pertpy facilitate standardized perturbation analysis (Heumos et al., 2026), and community perspectives are beginning to define the open benchmarking problems explicitly (Roohani et al., 2025; Luecken et al., 2025). The next step is to connect these resources into living benchmarks that are scenario-specific, versioned and difficult to game.

A practical benchmark commons for virtual cells should include standardized metadata for perturbation identity, dose, time, assay, donor, batch and context; predefined split families aligned to biological claims; strong simple baselines; and an evaluation suite spanning reconstruction, ranking, heterogeneity, calibration and context transfer. Benchmark reports should be accompanied by a minimal checklist stating what was held out, what support remained, which baseline was hardest to beat, how uncertainty was quantified and whether any external or orthogonal validation was performed. A leaderboard that ignores extrapolation, uncertainty and support structure is not a benchmark for trustworthy virtual cells. It is only a benchmark for interpolation. Table 2 summarizes a practical benchmark-design framework and a reporting checklist for trustworthy virtual-cell studies.

**TABLE 2:** Claim-matched reporting checklist for trustworthy virtual-cell studies.

Dimension	Key question	Minimum reporting elements	Common failure mode	Stronger evidence
Biological task	What biological decision is the model meant to support?	Explicit task definition and deployment claim	Reporting generic reconstruction scores for a decision-support claim	Task-linked endpoints such as perturbation ranking, pathway recovery or hit enrichment
Data support	Does the training data actually cover the intended counterfactual space?	Perturbation, dose, time, donor, batch and context metadata	Hidden support gaps or imbalanced design mistaken for generalization	Support diagnostics and explicit support limits
Split design	What is held out, and why does that match the claim?	Cell-, donor-, study-, perturbation- or context-level split description	Leakage through random cell holdout	Scenario-specific OOD splits aligned to deployment
Baselines	Has the method been compared against simple strong baselines?	Linear, nearest-neighbour or batch-aware baselines	Gains only versus weak neural comparators	Baseline suite with ablations
Metrics	Do metrics reflect the biological endpoint?	Gene-, pathway-, cell-state- and ranking-level metrics	Overreliance on global correlation or embedding scores	Multi-level evaluation with tail-risk summaries
Uncertainty	Does confidence track correctness?	Calibration or selective-prediction analysis	Point estimates presented as certainty	Confidence-error curves and abstention policies
Interpretability	What exactly is being explained?	Level of explanation, priors used, and test strategy	Post hoc saliency overinterpreted as mechanism	Program-level or regulator-level explanations with orthogonal support
Orthogonal validation	Has the claim been challenged outside the training readout family?	Independent assay type or external dataset	Transcriptome-only self-confirmation	Imaging, protein, chromatin, spatial or functional validation
Prospective testing	Has the model successfully guided new experiments?	Description of prospective selection rules and success criterion	Retrospective validation presented as discovery	Prospective hit enrichment or intervention selection
Update loop	Does validation inform model revision?	Error taxonomy and retraining logic	Validation is used only as an endpoint	Closed-loop workflow with support expansion

## Interpretability and mechanistic faithfulness

**FIGURE 5:**
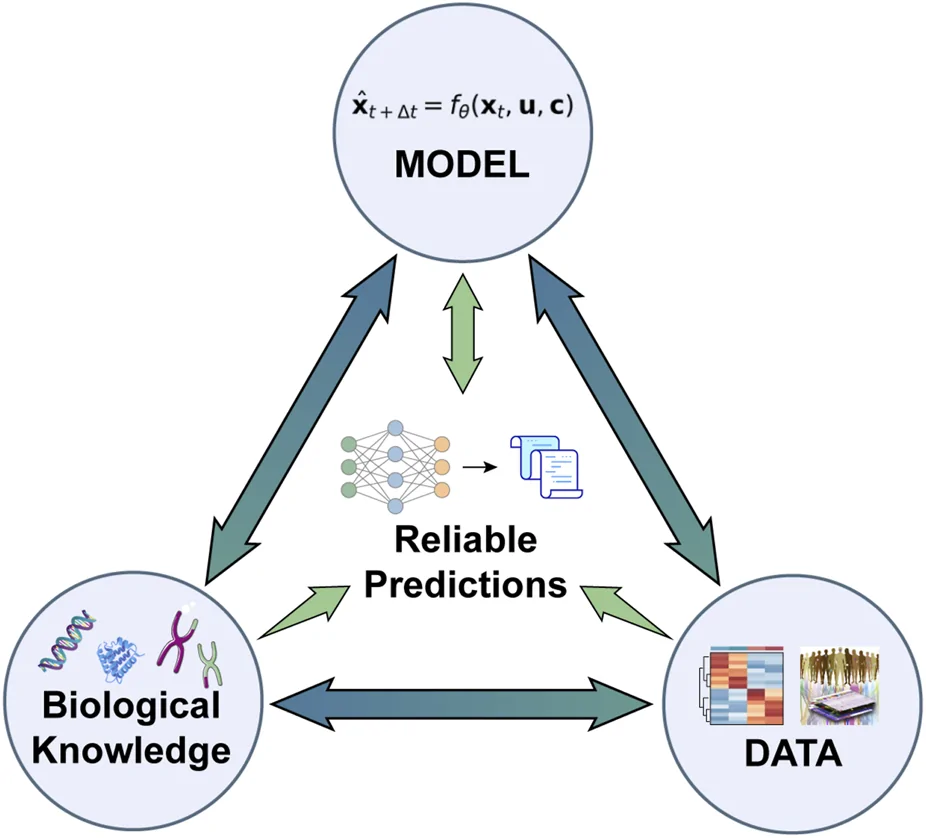
The faithfulness triangle for reliable virtual-cell prediction. Reliable predictions require consistency among the computational model, empirical data, and biological knowledge.

Interpretability is often invoked as a blanket virtue, but the more demanding question for virtual cells is narrower: what kind of explanation is required for a model to support mechanistic claims? A model can be operationally useful even if it is not fully transparent. Once its outputs are used to nominate regulators, prioritize interventions or justify causal narratives, however, the burden of explanation changes (Figure 5) (Wu, 2026; Yang et al., 2024; Theodoris et al., 2023; Noutahi et al., 2025; Boiarsky et al., 2024; Wang T. et al., 2025; Chevalley et al., 2025; He et al., 2025; Wang F-a et al., 2025; Li et al., 2025; Wu et al., 2025). Trustworthy virtual cells therefore require not only benchmarked performance but also evidence that the explanations attached to their predictions are biologically meaningful.

### Interpretability is multi-level and use-case dependent

Interpretability is not a single property. It can reside in the model class, in the learned representation, in the output object or in the validation strategy. Mechanistic models are interpretable because their variables and interactions correspond to explicit biological assumptions (Loew and Schaff, 2001; Karr et al., 2012; Johnson et al., 2023; Bunne et al., 2024). Deep generative and foundation models operate differently: they often learn internal states that are useful but not intrinsically legible (Bunne et al., 2024; Cui et al., 2024; Hao et al., 2024; Yang et al., 2024; Fu et al., 2025; Boiarsky et al., 2024). Neither regime is universally superior. What matters is whether the explanatory form is appropriate for the use case.

For virtual cells, it is useful to distinguish four levels of interpretability. Feature-level interpretability asks which genes, proteins or covariates influence a prediction. Representation-level interpretability asks whether latent spaces align with biologically meaningful manifolds such as cell types or trajectories. Program-level interpretability asks whether the model recovers coordinated regulatory modules, pathways or interaction programs. Counterfactual interpretability asks whether the explanation remains coherent when the model is queried under intervention: why this perturbation, in this context, produces this outcome rather than another. Higher levels bring the explanation closer to mechanistic usefulness.

### From feature attribution to regulatory programs

The simplest explanations in deep models are feature attributions, gene saliency, attention weights or local gradients. In single-cell data, correlated genes, sparse counts and batch structure can all produce unstable or misleading attributions. The problem is amplified in perturbation modeling, where a model may correctly forecast a response while assigning explanatory weight to genes that merely co-vary with the true drivers.

An important exception is a deeply sampled, dynamic perturbation setting in which a single modality captures the relevant response axis and attribution is followed by causal testing. ProteinTalks used large-scale perturbation proteomics and SHAP-based interpretation to nominate AKR1C3 as a resistance-associated target, and the proposed role was subsequently tested and supported by siRNA knockdown (Sun et al., 2025). This example does not make SHAP values mechanistic evidence by themselves; rather, it shows that feature attribution can become an actionable hypothesis generator when data support is unusually rich and wet-lab perturbation closes the causal loop.

More informative explanations arise when models recover programs rather than isolated features. Knowledge-informed and network-aware models aim to preserve regulatory or pathway structure in the representation (Yang et al., 2024; Theodoris et al., 2023; Li et al., 2025). Classical regulatory-network tools such as SCENIC, formal benchmarks for network inference, and *in silico* perturbation frameworks that explicitly reason about regulators all support the same lesson: explanations become more meaningful when they are expressed in biological objects that experiments can challenge (Aibar et al., 2017; Pratapa et al., 2020; Kamimoto et al., 2023). Methods such as CellNavi, scKAN and scTFBridge are notable because they move from generic feature importance toward intervention-relevant regulatory reasoning (Wang T. et al., 2025; He et al., 2025; Wang F-a et al., 2025).

A trustworthy virtual cell does not need to explain every molecular event, but it does need to expose enough of the relevant causal scaffold for the intended decision—for example, a driver gene, a transcription-factor module or a small set of pathways predicted to mediate rescue.

### Mechanistic faithfulness is stricter than *post hoc* plausibility

Interpretability becomes scientifically meaningful only when tied to mechanistic faithfulness. By this we mean that the biological narrative used to justify a prediction is constrained by intervention data and remains stable under tests that matter for the use case. A mechanistically faithful explanation need not be complete, but it should satisfy four criteria. First, it should be intervention-consistent: when a proposed driver is perturbed, the predicted direction of effect should hold. Second, it should be context-specific: the explanation should change when the relevant cell state or microenvironment changes. Third, it should be counterfactually stable: small perturbations to irrelevant features should not radically alter the narrative. Fourth, it should be externally challengeable through orthogonal assays.

These requirements are stricter than *post hoc* plausibility. A pathway label attached after prediction may sound convincing yet remain unsupported by causal evidence. Conversely, an explanation that survives perturbational, contextual and orthogonal challenge is scientifically valuable even if it is partial. The distinction is crucial because virtual-cell papers often drift from prediction to mechanism without upgrading the evidence base.

### Designing for faithfulness: priors, constraints and compositionality

Mechanistic faithfulness is easier to assess when it is partly designed into the model. Priors, graph constraints, compositional decompositions and modular latent structures can all help tie representation to biological meaning (Yang et al., 2024; Theodoris et al., 2023; Wang T. et al., 2025; Chevalley et al., 2025; He et al., 2025; Wang F-a et al., 2025; Li et al., 2025; Bahrami et al., 2026). Such design choices do not guarantee valid mechanism, but they can make unsupported explanations less likely and more testable. They also align with a broader shift in the field away from purely modality-specific models toward compositional, knowledge-grounded systems (Bahrami et al., 2026).

The future of trustworthy virtual cells is therefore unlikely to be a choice between “fully interpretable” and “fully black-box” models. More plausible are architectures in which some components are learned flexibly, whereas others are deliberately structured for regulatory interpretation, uncertainty propagation and modular validation.

### A practical reporting framework for interpretability claims

Interpretability claims should be reported with the same discipline as predictive benchmarks. At minimum, authors should state what is being explained, at which level of abstraction, with what stability across seeds or datasets, and against which external evidence. If a model claims to identify drivers, those drivers should be compared with perturbational or orthogonal evidence where possible. If it claims pathway recovery, the pathway object and scoring procedure should be explicit. If the explanation is purely *post hoc*, that limitation should be acknowledged.

## Closed-loop validation: from prediction to falsification

**FIGURE 6:**
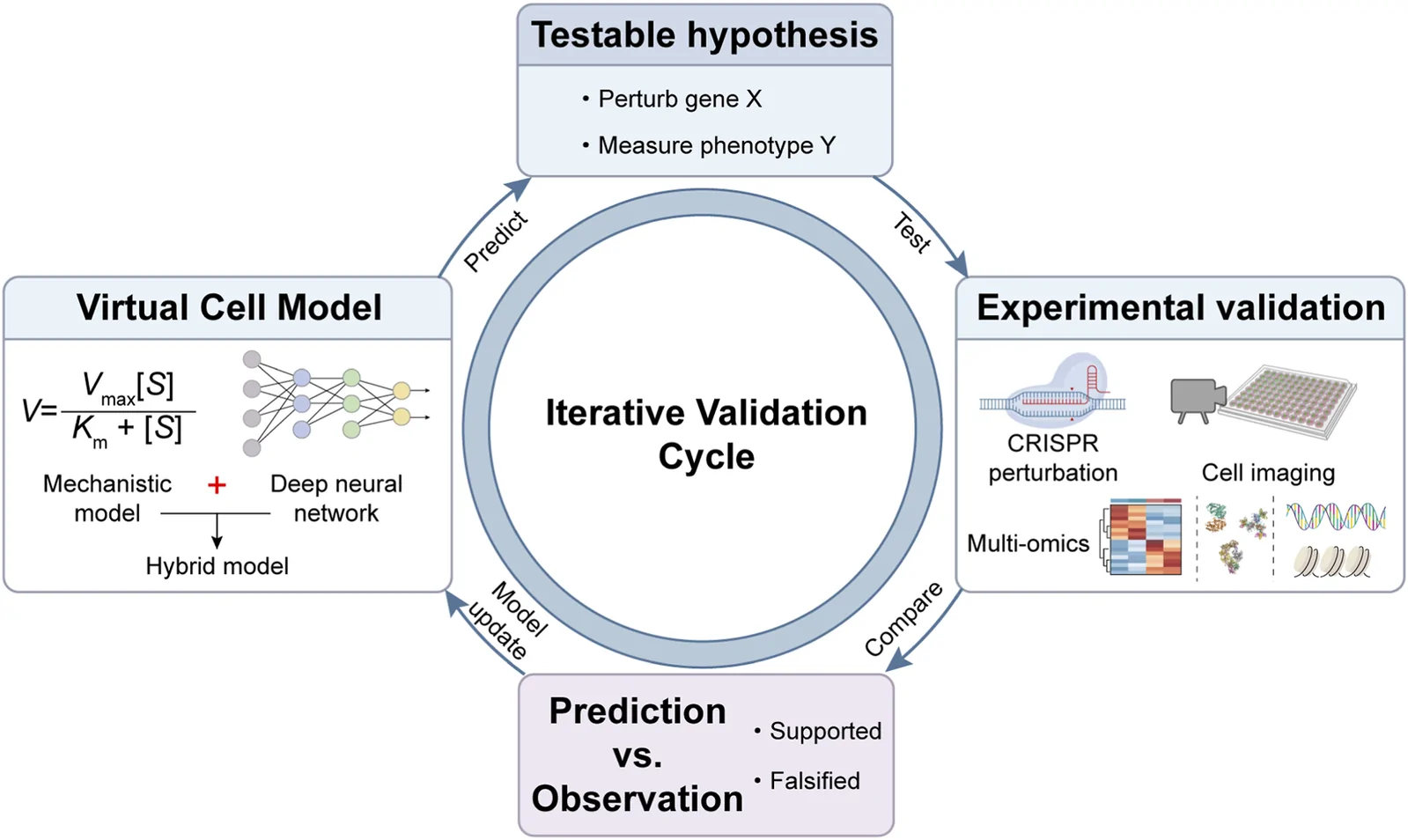
Closed-loop validation of virtual-cell models. Model-generated hypotheses are experimentally tested using perturbation and orthogonal assays. Comparison between predicted and observed outcomes supports or falsifies hypotheses and feeds back into iterative model refinement.

Validation is the point at which virtual cells become scientific instruments rather than sophisticated predictors. The decisive question is not whether a model reproduces held-out data from the same measurement pipeline, but whether its outputs survive challenge by experiments that were not already encoded in the training objective (Ma et al., 2026; Qian et al., 2025; Roohani et al., 2025; Wu, 2026; Chandrasekaran et al., 2024; Dixit et al., 2016; Srivatsan et al., 2020; Feldman et al., 2022; Kudo et al., 2025; Wei et al., 2026; Zheng et al., 2025; Replogle et al., 2022; Gangwal and Lavecchia, 2025). For this reason, validation should be conceived as a ladder rather than a binary event (Figure 6).

### Validation is a ladder, not a binary event

Different deployment claims require different levels of evidence. A model used for annotation or imputation may need only strong internal validation. A model used to nominate regulators or rank interventions requires orthogonal challenge. A model used to prioritize disease-modifying strategies or reduce experimental burden in preclinical programs requires stronger external evidence, ideally across assay systems (Ma et al., 2026; Wu, 2026; Zheng et al., 2025; Gangwal and Lavecchia, 2025).

Thinking in terms of a validation ladder helps prevent category errors. It is reasonable for a paper to claim that a model improves perturbation forecasting within a defined assay setting. It is not reasonable to infer from that alone that the same model is ready for mechanistic discovery or translational triage. The validation ladder should rise with the ambition of the claim.

### CRISPR and perturbation sequencing are the primary falsification engines

For cell-intrinsic mechanisms, pooled perturbation sequencing remains the primary falsification platform (Dixit et al., 2016; Replogle et al., 2022; Datlinger et al., 2017; J et al., 2016; Adamson et al., 2016; Schraivogel et al., 2020; Replogle et al., 2020). Perturb-seq and related methods allow model predictions about regulators, combinations or pathways to be tested directly in cellular populations. They are especially powerful because they can be run iteratively: the model nominates hypotheses, targeted screens challenge them, and the resulting discrepancies are used to refine both architecture and training data (Dixit et al., 2016; Replogle et al., 2022).

This iterative logic is central to closed-loop learning. Sequential optimal experimental design has already been proposed for perturbation screens guided by multimodal priors (Bunne et al., 2024; Qian et al., 2025; Roohani et al., 2025; Rood et al., 2024a). Community visions of the virtual cell similarly emphasize an experimental loop in which prediction and data generation co-evolve (Bunne et al., 2024; Qian et al., 2025; Roohani et al., 2025; Rood et al., 2024a). In this framework, validation is not the end of the pipeline. It is a source of new information about where the model’s support boundary actually lies.

### Model-organism and automated platforms as an intermediate rung

Fully automated loops in human organoids or microphysiological systems remain difficult because these assays are costly, slow and sparsely sampled. Genetically tractable model organisms coupled to robotic experimentation and active learning offer a practical intermediate rung between cell-line screens and human tissue models. The virtual-yeast framework in *Saccharomyces cerevisiae* demonstrates how multimodal data, mechanistic modules and autonomous experiment design can be coordinated in a closed-loop platform (Qian et al., 2026). Such systems provide a lower-cost environment for debugging acquisition functions, uncertainty policies and update rules before escalation to human tissues.

### Orthogonal phenotypes prevent transcriptome self-confirmation

Transcriptomic validation alone is often insufficient because it risks confirming the model in the same modality from which it learned. Orthogonal phenotypes—morphology, protein readouts, functional assays or viability—provide a stronger challenge (Chandrasekaran et al., 2024; Feldman et al., 2022; Kudo et al., 2025; Bray et al., 2016; Way et al., 2022). Image-based pooled screens are particularly useful because they scale and because morphology often captures integrated cellular consequences of perturbation that are not obvious in transcriptomics alone (Chandrasekaran et al., 2024; Feldman et al., 2022; Kudo et al., 2025).

This principle extends beyond imaging. Proteomic assays, pathway activity reporters, electrophysiology and simple phenotypic endpoints such as proliferation or death can all serve as external stress tests. The exact phenotype matters less than the logic: a trustworthy virtual cell should be able to survive challenge from a measurement channel that is not trivially entangled with its training target.

### Escalating validation into organoids, microphysiological systems and tissue context

Cell-intrinsic assays cannot validate every claim. Once the target question concerns tissue patterning, disease progression, immune interactions or translational response, validation must be escalated into richer biological systems (Kudo et al., 2025; Zheng et al., 2025; Kaul et al., 2023; Gangwal and Lavecchia, 2025; Low et al., 2021; Kim et al., 2020; Fatehullah et al., 2016). Organoids and organs-on-chips are particularly attractive because they preserve more native architecture and can be perturbed systematically (Low et al., 2021; Kim et al., 2020; Fatehullah et al., 2016). They are not simple substitutes for *in vivo* biology, but they are valuable intermediate systems for testing whether a model’s conclusions survive greater contextual complexity.

Recent disease-oriented generative modeling work illustrates this trajectory. Models such as UNAGI attempt to infer disease dynamics and nominate therapeutic hypotheses, then subject those predictions to downstream experimental challenge (Zheng et al., 2025). This is a useful template for the field: action-oriented virtual cells should be validated in systems whose biological resolution matches the deployment claim.

### Error analysis should update the model, not merely qualify the paper

Validation failures are often treated as caveats. They should instead be treated as data. If a predicted perturbation effect fails in a particular lineage, dose range or tissue context, the discrepancy reveals something about both the biological system and the model’s support boundary. Closed-loop virtual-cell research should therefore include explicit error analysis: which assumptions failed, which inputs were missing, which priors were too rigid and which contexts were underrepresented?

The best experimental companions to virtual-cell models are not generic validation assays; they are assays selected because they are maximally informative about uncertainty, mechanism or support gaps. The validation loop should therefore be part of model design from the start.

### Evidence requirements should match the deployment claim

A simple rule follows from the validation ladder: the more action-oriented the claim, the stronger the evidence required. Claims about state reconstruction need not be validated like claims about drug prioritization. Claims about exploratory mechanism need not meet the same standard as claims about reducing preclinical burden. This proportionality principle should be stated explicitly in virtual-cell papers, because much of the current confusion in the field comes from mixing the language of decision support with the evidence base of interpolation.

A trustworthy virtual cell is therefore not defined by having passed one validation assay. It is defined by having been challenged at an evidence level appropriate to its intended use and by having incorporated those challenges into model refinement.

## From trustworthy models to decision-grade applications

The most compelling promise of virtual cells is not that they will eliminate experiments, but that they will make experiments more selective, more mechanistically grounded and more informative (Ma et al., 2026; Wu, 2026; Zheng et al., 2025; Bao et al., 2025; Gangwal and Lavecchia, 2025). In the near term, three application classes appear especially realistic.

The first is target and regulator prioritization, where models benchmarked under unseen perturbations can nominate candidates for focused follow-up rather than broad screening (Roohani et al., 2024; Wei et al., 2026; Replogle et al., 2022; Wang T. et al., 2025). The second is mechanism-of-action and drug ranking, where perturbation transcriptomics, morphology and pathway priors can help cluster programs and prioritize rescue experiments (Chandrasekaran et al., 2024; Srivatsan et al., 2020; Zheng et al., 2025; Bao et al., 2025; Lamb et al., 2006; Subramanian et al., 2017; Wishart et al., 2018). The third is experimental triage in preclinical systems, where the model helps decide which doses, combinations, genetic contexts or organoid models are worth testing first (Ma et al., 2026; Zheng et al., 2025; Gangwal and Lavecchia, 2025; Low et al., 2021; Kim et al., 2020; Fatehullah et al., 2016; Björnsson et al., 2020).

Concrete examples remain proof-of-principle rather than evidence of autonomous translation. ProteinTalks linked proteomic drug-response prediction to AKR1C3 nomination and siRNA testing (Sun et al., 2025); UNAGI connected disease-state modeling to therapeutic hypothesis generation and downstream challenge (Zheng et al., 2025); and the virtual-yeast programme couples model predictions to active experimentation in a tractable eukaryotic system (Qian et al., 2026). Together, these cases show meaningful influence on target selection and experiment design while stopping short of decision-exclusive clinical use.

Across all three classes, the correct near-term framing is decision-grade support, not full autonomy. A virtual cell becomes decision-grade when its forecasts are reliable enough to improve the ordering of experiments. It becomes decision-exclusive only when it can justify action without substantive external validation. The field is not there yet.

This distinction is particularly important for translational rhetoric. Terms such as digital twin, patient avatar or *in silico* trial can be useful organizing metaphors (Gangwal and Lavecchia, 2025; Björnsson et al., 2020), but they can also encourage overclaiming if not tethered to evidence. For most current systems, the realistic contribution is earlier and narrower: reducing search space, linking candidate mechanisms to perturbational evidence, and exposing which experimental next steps are most informative. That is already a substantial advance.

## Limitations and challenges

Three limitations dominate current virtual-cell research. First, perturbation data remain short-term, RNA-centric and concentrated in a narrow set of cell lines, with sparse coverage of dose-response surfaces, sequential interventions and long-term adaptation (Peidli et al., 2024; Dixit et al., 2016; Srivatsan et al., 2020; Wei et al., 2026; Replogle et al., 2022; Ahlmann-Eltze et al., 2025). Second, matched datasets that jointly capture perturbation, time, donor diversity and spatial or multicellular context are rare, limiting generalization to immune modulation, tissue remodeling and disease progression (Klein et al., 2025; Biancalani et al., 2021; Tejada-Lapuerta et al., 2025; La Manno et al., 2018; Bergen et al., 2020; Schiebinger et al., 2019; Setty et al., 2019; Wolf et al., 2019; Longo et al., 2021; Dries et al., 2021; Palla et al., 2022; Browaeys et al., 2020; Jin et al., 2021). Third, inconsistent metadata, non-standard benchmark splits and the cost of large multimodal models impede reproducibility and equitable access (Peidli et al., 2024; Heumos et al., 2026; Luecken et al., 2022; Luecken et al., 2025; Fu et al., 2025; Boiarsky et al., 2024; Zeng et al., 2025; Kalfon et al., 2025; Kedzierska et al., 2025; Bahrami et al., 2026).

Mechanistic interpretation and closed-loop validation add a second layer of difficulty. Saliency maps or latent directions often lack intervention consistency, and orthogonal assays differ markedly in throughput and cost (Chandrasekaran et al., 2024; Feldman et al., 2022; Kudo et al., 2025; Yang et al., 2024; Zheng et al., 2025; Theodoris et al., 2023; Gangwal and Lavecchia, 2025; Boiarsky et al., 2024; Kedzierska et al., 2025; Wang T. et al., 2025; Chevalley et al., 2025; He et al., 2025; Wang F-a et al., 2025; Li et al., 2025; Wu et al., 2025; Bahrami et al., 2026; Aibar et al., 2017; Pratapa et al., 2020; Kamimoto et al., 2023; Low et al., 2021; Kim et al., 2020; Fatehullah et al., 2016). Accordingly, the criteria proposed here should be applied proportionally: compact models with internal validation may be adequate for bounded tasks, whereas action-oriented claims require uncertainty calibration and prospective or orthogonal testing. Current systems therefore satisfy different subsets of the framework and should generally be viewed as decision-grade support rather than decision-exclusive tools.

## Outlook: standards, benchmark commons and the path to virtual tissues

Addressing the limitations outlined above will require progress on three priorities. The first is benchmark commons: shared, versioned evaluation resources with leakage-resistant splits, strong baselines and scenario-specific metrics. The second is structured model design: architectures that combine scalable learning with biologically meaningful priors, modularity and explicit uncertainty. The third is validation standards, under which the community expects the strength of orthogonal and external validation to rise with the ambition of the claim.

If these priorities are met, the field can move from single-task predictors toward more integrated virtual tissues and disease systems. If they are not, virtual-cell research risks becoming a succession of increasingly large models evaluated on increasingly permissive benchmarks. The decisive transition is not from small models to large ones, but from predictive virtual cells to trustworthy virtual cells. The trustworthy virtual cell is therefore best understood today as an evaluative and developmental target rather than a claim that a fully integrated system already exists. Current models instantiate individual components, including representation learning, perturbation forecasting, calibration, mechanistic modules and experimental feedback, at different levels of maturity.
